# 老年非小细胞肺癌生存者运动行为改变的研究

**DOI:** 10.3779/j.issn.1009-3419.2010.01.12

**Published:** 2010-01-20

**Authors:** 红艳 应, 毓洲 王, 晓红 宁, 建凤 周, 林 赵, 亚娟 邵, 春梅 白, 书长 陈

**Affiliations:** 100730 北京，中国医学科学院北京协和医学院北京协和医院肿瘤内科 Department of Medical Oncology, Peking Union Medical College Hospital, Peking Union Medical College, Chinese Academy of Medical Sciences, Beijing 100730, China

**Keywords:** 肺肿瘤, 恶性肿瘤生存者, 老年, 体能锻炼, Lung neoplasms, Cancer survivor, Elderly, Physical acitivity

## Abstract

**背景与目的:**

运动对健康有重要影响，可以提高癌症生存者的心血管系统功能、肌力和幸福感等，并减少乏力、焦虑、抑郁等。但目前关于肺癌患者治疗后运动能力的改变及其与肿瘤复发的关系目前尚不明了。本研究拟探讨老年非小细胞肺癌生存者体能锻炼行为模式的改变和临床意义。

**方法:**

采用回顾分析方法，调查包括手术后、化放疗后病情稳定者和服用酪氨酸激酶抑制剂行靶向治疗的老年肺癌生存者，记录其诊断前、积极治疗3个月和1年的运动量和每周运动频率，了解患者运动动机和偏好，探讨运动与肿瘤复发的关系。

**结果:**

调查了58例老年肺癌生存者，发现治疗期间老年肺癌生存者的体能锻炼参与比例下降，但1年后平均轻度体力活动较治疗前有增加。1年后运动基本达标的患者占75.9%（44/58），肺癌复发/进展率为20.0%（7/35），不达标的患者占24.1%（14/58），复发/进展率为35.7%（5/14），运动不达标时肿瘤复发/进展的风险比（risk ratio, RR）为2.14（95%CI:0.81-5.68, *P*=0.26）。肿瘤生存者运动的动机主要有改善健康、增加体能，维持正常生活方式、增加免疫力等，而最常见的障碍是疲乏、不舒服和缺少动机等。

**结论:**

治疗期间老年肺癌生存者的体能锻炼参与比例降到原有的10%以下，而且即便是治疗完成后也不能恢复到诊断前的水平，运动不达标时和运动达标时患者肿瘤复发/进展的情况无明显差异，运动与肿瘤复发的关系有待进一步研究。

## Abstract (en)

**Background and objective:**

Exercise can improve circulation, muscular strength and happiness of cancer survivors. But more data were needed to demonstrate both the exercise ability of cancer suivivors after pulmonary lobectomy and the influences of exercise on their survivals. The aim of this study was to examine changes of exercise and its clinical effects among eldly non-small cell lung cancer survivors.

**Methods:**

Elderly non-small cell lung cancer survivors who had progression-free disease after surgery, chemotherapy, radiation therapy or tyrosine kinase inhibitors were included. Their exercises and participation rates per week before cancer diagnosis, after 3 months anticancer therapy and 1 year after diagnosis as well as their exercise motivations and prevalences were investigated retrospectively.

**Results:**

Forty-eight elderly non-small cell lung cancer survivors were selected. Moderate-vigorous intensity exercise had by the elderly progressin-free non-small cell lung cancer survivors after diagnosis decreased, but the participation rate of light intensity exercise was higher in 1 year after diagnosis than before diagnosis. 75.9% (14/58) patients had exercise up to the standard and the cancer recurrence rate was 20.0% (7/35). The recurrence rate of the other group was 35.7% (5/14), and the risk ratio of recurrence was 2.14 (95%CI: 0.81-5.68, *P*=0.26). The most common motivations of exercise were improving health, increasing physical activity, maintaining healthy life style and improving immunity. And the main disturbances were fatigue, discomfort and lack of motivation.

**Conclusion:**

The exercise participation rate during anticancer treatment among the elderly non-small cell lung cancer survivors decreased and did not return to prediagnosis levels after treatments were completed. The relationship between exercise and recurrence of cancer was not clear and needed further work.

体能锻炼的运动刺激和行为在任何人群中都是一种重要的需求，但是在有慢性疾病的人群中，比如癌症生存者，这种需求存在很多问题。一些肿瘤生存者经常忍受长期艰难的药物治疗，而对运动的潜在好处和长远益处并不清楚，这将使癌症生存者参与运动更加困难。肺癌是目前恶性肿瘤中最常见、致死率最高的疾病，也是老年人常见的恶性肿瘤。据统计，50%以上的晚期非小细胞肺癌(non-small cell lung cancer, NSCLC)患者诊断时年龄超过65岁。随着年龄的增长，人体的器官功能和治疗药物的药代动力学等都随之改变，而老年肺癌患者经过胸部手术、放化疗等治疗后，身体机能会发生改变，由于老年人心肺代偿功能差，这些肿瘤生存者的体能锻炼有其特殊性。因此，我们采用回顾性分析方法，调查老年肺癌生存者诊断前、治疗中和治疗后的运动形式和运动强度的变化，对患者运动动机进行考察，并探讨运动与肿瘤复发之间的关系，探讨这一领域将来的重要的研究方向，为老年肺癌生存者运动与健康指导提供依据。

## 1 对象和方法

### 1.1 对象

回顾性调查我科门诊和住院治疗的70岁以上老年NSCLC生存者，包括手术后、化放疗后病情稳定者和服用酪氨酸激酶抑制剂靶向治疗的患者。排除标准为有承重骨转移或者脑转移者、脑血管意外有后遗症者、严重心肺疾病的患者或其它不适合运动的情况。最终纳入研究病例共58例，见表 1。

**1:** 患者临床特征（*n*=58） Patient characteristics (*n*=58)

Characteristics	*n*
No. of patients	58
Sex	
Male	39 (62%)
Female	19 (38%)
Disease stage at diagnosis	
Ⅰ	9 (15.5%)
Ⅱ	24 (41.4%)
Ⅲ	18 (31.0%)
Ⅳ	7 (12.1%)
Prior therapy for disease	
Surgery	13 (22.4%)
Surgery+Chemotherapy	16 (27.6%)
Surgery+Chemotherapy+Chest radiotherapy	6 (10.3%)
Surgery+Chest radiotherapy	4 (6.9%)
Surgery+TKI	3 (5.1%)
Chest radiotherapy+TKI	2 (3.4%)
Chemotherapy+Chest radiotherapy	8 (13.8%)
Chemotherapy+TKI	5 (8.6%)
TKI	1 (1.7%)

### 1.2 运动形式和比例调查

采用由美国运动医学学院和美国心脏医学会推荐的运动方案^[1]^，记录患者在诊断之前、积极治疗期间和治疗完成后的轻、中、高强度运动的频率。高强度运动为每次连续20 min -30 min的慢步长跑或者类似强度的其它体能活动，中等强度运动为一次30 min或更长时间的运动(比如慢步走路)。轻度运动为不足30 min的慢步走路。运动频率以每周运动次数计算，调查肿瘤生存者的运动动机、运动的主要好处与运动受限的因素。

### 1.3 运动量达标状况与肿瘤复发或进展的关系

本调查中，肿瘤进展定义为晚期肿瘤患者经过化放疗和靶向等综合治疗后，病情相对稳定，但在随诊评估过程中参照RECIST标准证实的疾病进展。肿瘤复发定义为早期肿瘤患者经过手术完全切除肿瘤后经过辅助治疗在随访过程中出现新发病灶。根据美国运动医学学院和美国心脏医学会^[1]^的标准，成人一周要有3 d进行20 min以上的高强度运动(比如慢步长跑)，或者一周有5 d进行30 min以上的中等强度的运动(比如慢步走路)，为运动达标。本研究对运动量达标与未达标的人群肿瘤复发或者疾病进展进行分析，探讨运动量未达标肺癌生存者疾病进展或复发的风险。

### 1.4 统计分析

本研究统计分析采用EPI info 3.5.1公共健康数据与统计专业软件，率的比较采用卡方检验和风险评估等方法进行数据统计和处理，以*P* < 0.05为有统计学差异。

## 2 结果

### 2.1 人口学特点

本研究中58例肺癌患者的人口学和临床特征见表 1。大多数患者为腺癌病例，占89.7%(52/58)，42例(73%)患者有肺部手术史(肺叶切除37例，侧肺切除3例，肺段切除2例)；平均年龄73.0岁，范围为70岁-82岁；平均PS评分为80分，范围为60分-100分。

### 2.2 癌症生存者的运动形式和疾病复发比例

在我们的调查中，记录了58例老年肺癌生存者诊断前、积极治疗3月和1年之间的运动量和每周运动频率，发现诊断肺癌后患者的运动基本停止，只有6例患者仍如常进行锻炼，为原来的9.4%(6/58)。患者经治疗病情平稳后进行中、高强度运动频率仍然比患者诊断之前低(图 1)，尤以治疗3个月时为著。但1年后患者进行轻度体力活动平均频率较治疗前有增加。根据美国运动医学学院和美国心脏医学会标准，1年后运动基本达标的患者占75.9%(44/58)，其肿瘤复发率为20%(7/35)；运动不达标患者占24.1%(14/58)，肿瘤复发率为35.7%(5/14)；运动不达标患者肿瘤复发和运动达标患者肿瘤复发的风险比(risk ratio, RR)为2.14(95%CI: 0.81-5.68, *P*=0.26)。

**1:**
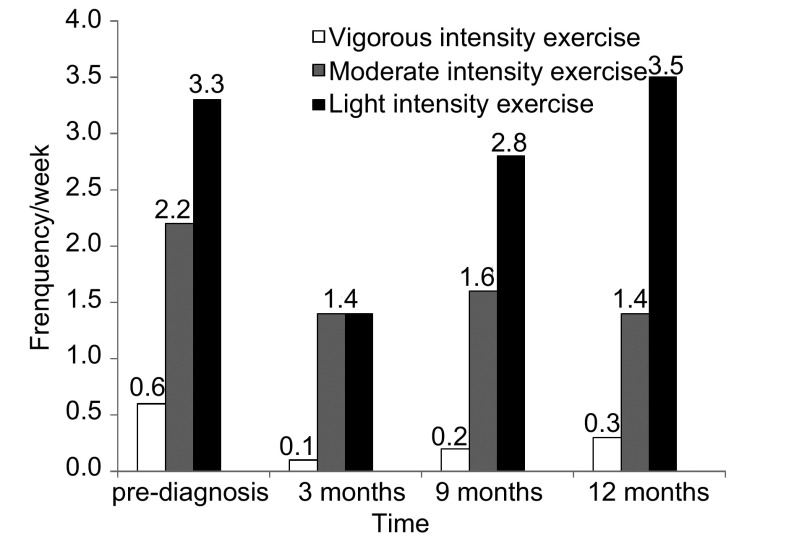
老年肺癌幸存者运动强度与运动频率变化 Changes in exercise frequency of elderly non-small cell lung cancer survivors

### 2.3 肺癌生存者运动动机和障碍分析

在对肺癌生存者的调查中，规定每个调查者选择运动动机和障碍10项中的5项，根据肺癌生存者对运动动机(主观认定的好处)和障碍(有运动要求，但不能进行运动的原因)选择结果，排列顺序见表 2。本研究中，5个常见的激发肿瘤生存者进行运动的动机是运动可以使感觉更好和改善健康，增加体能、维持正常生活方式，增加免疫力，从手术和治疗中恢复以及控制癌症、防止复发。5个常见的运动障碍是：没有力气/疲乏、感觉不舒服、缺少动机/懒惰、恶心等化疗反应和疼痛。

**2:** 老年肺癌幸存者运动强度与运动频率变化 Changes in exercise frequency of elderly non-small cell lung cancer survivors

	Exercise motives	Percent (%)	Exercise barriers	Percent (%)
1	Feel beter and improve well-being	89.7	Fatigue/tiredness	75.9
2	Increase energy and maintain a normal life style	86.2	Malaise or feeling ill	63.8
3	Improve their immune function	82.8	A lack of motivation/laziness	56.9
4	Recover from surgery and treatment	72.4	Nausea	50.0
5	Gain control over cancer	60.3	Pain or soreness	48.3
6	Keep in shape and control weight	44.8	No counseling for exercise	46.6
7	Cope with the stress of cancer and treatment	25.9	Worring to be injured	44.8
8	Muscular strength	19.0	Lack of place and facilities	44.8
9	Improve circulation and inspiration	12.1	Lack of time	36.2
10	Get mind off cancer and treatment	6.9	Not approved by familly	32.8

## 3 讨论

运动对健康有重要意义，研究表明，运动可以提高癌症生存者的心血管系统功能、肌力、自尊心和幸福感，减少乏力、焦虑、抑郁等^[2-4]^，即使是进展期行姑息治疗的患者，运动也能提高其生活质量^[5]^。目前关于肺癌患者经过手术、放化疗后，其运动能力的改变和健康关系的研究报道尚少。本研究的对象为老年肺癌生存者，采用由美国运动医学学院和美国心脏医学会推荐的指南，对其运动强度和频率进行初步评估。指南推荐成人一周要有3 d进行连续20 min-30 min的重强度运动(比如慢步长跑)，或者一周有5 d进行累积30 min或更长时间的中等强度的运动(比如慢步走路)。尽管这些指南可能适合已经完成初步治疗、被认为是摆脱疾病的癌症生存者，但是它们是否适合肺部有病变或者进行肺癌手术的老年癌症生存者还不太清楚。因此，采用这一评估方法，评估老年肺癌生存者诊断前、治疗期间和治疗后的运动强度和频率，探讨肿瘤生存者的特殊人群-老年肺癌生存者的运动模式，对指导肿瘤生存者运动有重要意义。以往研究对老年肿瘤生存者、年轻肿瘤生存者和其他非肿瘤人群的运动模式是否存在差异尚不清楚，Coups等^[6]^对包括超过1 600名癌症生存者在内的320 000成人中的2 000人进行了抽样自然健康面试调查，在年轻(18岁-39岁)和年老(> 65岁)的两个同龄组中，癌症生存者与非癌症对照组的运动参与比例并没有差别，但是在中段年龄组(40岁-64岁)，两组患者运动参与比例有明显差别，非癌症对照组体能锻炼达标率约31%，而癌症生存者中只有25%患者进行体能锻炼。文献^[7-9]^表明，癌症生存者在接受抗癌治疗时运动水平明显降低；治疗之后患者运动水平会有一定的恢复，但通常不能达到诊断之前的水平，这部分与抗癌治疗造成患者心肺、神经和肌肉系统的不良反应有关。且患者的活动能力受患者肿瘤类型、治疗手段和生存时间的影响^[10]^而不一致。我们调查的老年肺癌生存者，在肿瘤治疗期间(也称为肿瘤生存者急性期)，其体能锻炼参与比例明显下降，在治疗完成后，患者中高强度体能锻炼活动均明显减少，但轻度体能锻炼(主要是散步)达标率占75.9%，这些老年肺癌生存者可以进行日常的活动。这与西方的研究报道不完全一致，可能与文化差异或者病例选择有关。

体能锻炼与肿瘤复发的关系目前尚不明了，但研究表明缺乏运动、肥胖和高代谢综合症人群的肿瘤复发机率增加，与胰岛素样生长因子及复杂的内分泌调节有关^[11]^。有一些资料^[12-15]^表明，体能锻炼可降低癌症生存者的复发率及癌症特异死亡率。一项包括3 000例乳腺癌患者的调查研究^[15]^发现，治疗完成后患者进行高水平的运动可以使乳腺癌的复发率、乳腺癌相关死亡率及总死亡率降低26%-40%；患者每周进行1 h -3 h的运动即可使复发率和死亡率下降，而在每周运动3 h -5 h的患者中，下降趋势更为明显。目前关于肺癌患者经过胸部手术、放化疗后，其运动能力的改变和健康关系的研究报道尚少，有2个小型研究通过对进展期NSCLC患者的饮食干预延长了患者的生存时间^[16, 17]^。本组运动不达标的患者，其肿瘤复发/进展率较运动达标的患者升高，其RR为2.14，但两组之间尚无统计学差异。由于本组病例数较少，是回顾性调查研究，需要进一步前瞻性的研究来证实。

以往调查发现，大部分(68%)癌症患者认为，锻炼是有益处的，但即使是这样，78%的患者并没有能够像他们希望的那样锻炼，不同肿瘤生存者的运动动机和障碍有一定的共同性^[18, 19]^。但是，体能运动的动机和障碍随患者治疗阶段而异，也可能与肿瘤的类型和治疗方式有关。化疗期间的运动障碍经常与治疗的不良反应如恶心、腹泻、乏力、抑郁等有关，而治疗之后的运动障碍与一般人群的运动障碍往往是一致的，如没有合适的锻炼机会、没有时间、太忙等。有调查发现，减轻体重是子宫内膜癌生存者最常见的运动动机，而没有条件运动是非霍奇金淋巴瘤淋巴瘤生存者主要的运动障碍。对于那些正在接受治疗的生存者而言，运动动机主要是运动会帮助他们应对他们的治疗、忘记他们的癌症和治疗的不良反应、维持正常的生活方式、减轻乏力和使他们保持强壮和健康等^[20]^。对于那些已经完成治疗的人，体能锻炼会减少他们癌症复发的风险和其它慢性疾病、提高免疫功能、提高能量水平与生活质量和回到正常的生活方式^[21]^，这与我们的调查结果一致。但是，关于癌症生存者运动动机和行为改变的研究目前尚少，尤其是针对老年癌症生存者。而且，研究癌症生存者的运动行为参数，例如需氧量、肌肉力量和功能等是有用的。为深入这些研究，应进行前瞻性研究设计并提高数据质量，对不同类型的肿瘤如卵巢癌、膀胱癌、肺癌的生存者进行比较，收集肿瘤生存者不同阶段(例如治疗前、不同治疗中、治疗后、长期生存)的数据。理论上，这些数据将是以人群为基础的，应与从一般人群和/或其它慢性疾病人群(例如糖尿病、心脏病)中得到的数据相比较，才会对因癌症经历而改变的运动行为的自然史有更好的理解。

综上所述，癌症生存者运动动机和行为改变的研究是对癌症生存者身心健康有重要意义的调查领域，目前关于这方面的研究尚少，但逐渐受到广泛关注。本组在运动未达标的患者，其肿瘤复发/进展率较运动达标的患者升高，RR为2.14，但尚无统计学差异。初步的研究已表明肿瘤治疗期间(也称为肿瘤生存者急性期)癌症生存者的体能锻炼参与比例明显下降，而且即便是治疗完成后也不能恢复到诊断前的水平。迄今尚缺乏以行为改变进行干预，证明会影响癌症生存者的肿瘤进程的数据，有待进一步研究。
